# Regulation of prefrontal cortex myelination by the microbiota

**DOI:** 10.1038/tp.2016.42

**Published:** 2016-04-05

**Authors:** A E Hoban, R M Stilling, F J Ryan, F Shanahan, T G Dinan, M J Claesson, G Clarke, J F Cryan

**Affiliations:** 1 APC Microbiome Institute, University College Cork, Cork, Ireland; 2 Department of Anatomy and Neuroscience, University College Cork, Cork, Ireland; 3 Department of Microbiology, University College Cork, Cork, Ireland; 4 Department of Psychiatry and Neurobehavioural Science, University College Cork, Cork, Ireland

## Abstract

The prefrontal cortex (PFC) is a key region implicated in a range of neuropsychiatric disorders such as depression, schizophrenia and autism. In parallel, the role of the gut microbiota in contributing to these disorders is emerging. Germ-free (GF) animals, microbiota-deficient throughout life, have been instrumental in elucidating the role of the microbiota in many aspects of physiology, especially the role of the microbiota in anxiety-related behaviours, impaired social cognition and stress responsivity. Here we aim to further elucidate the mechanisms of the microbial influence by investigating changes in the homeostatic regulation of neuronal transcription of GF mice within the PFC using a genome-wide transcriptome profiling approach. Our results reveal a marked, concerted upregulation of genes linked to myelination and myelin plasticity. This coincided with upregulation of neural activity-induced pathways, potentially driving myelin plasticity. Subsequent investigation at the ultrastructural level demonstrated the presence of hypermyelinated axons within the PFC of GF mice. Notably, these changes in myelin and activity-related gene expression could be reversed by colonization with a conventional microbiota following weaning. In summary, we believe we demonstrate for the first time that the microbiome is necessary for appropriate and dynamic regulation of myelin-related genes with clear implications for cortical myelination at an ultrastructural level. The microbiota is therefore a potential therapeutic target for psychiatric disorders involving dynamic myelination in the PFC.

## Introduction

In mammals, the prefrontal cortex (PFC) is essential in emotional learning, has a prominent role in fear and anxiety processes and has been implicated in the regulation of the hypothalamic–pituitary–adrenal axis.^1, 2^ Along with the amygdala, the PFC comprises a central neuronal circuit underlying emotional regulation^3^ and also facilitates memory storage, behavioural flexibility and attention. The PFC is thus a very dynamic brain region, and its dysfunction is deeply implicated in many neuropsychiatric and neurodevelopmental disorders such as schizophrenia and autism spectrum disorders.^4, 5^ Over the past decade, a growing body of preclinical studies have highlighted the prominent role of the gut microbiota in gut–brain interactions.^6, 7^ Disturbance in this bidirectional route of communication has relevance for both gastrointestinal and brain disorders,^8^ including autism spectrum disorders,^9^ Parkinson's disease^10^ and even demyelinating disorders of the central nervous system (CNS),^11, 12^ and manipulating gut microbial diversity by administration of antibiotics,^13, 14^ probiotics^15^ and prebiotics^16^ has shed light on their potential to modulate brain and behaviour. In addition, proof-of-principal experiments using microbiota-deficient germ-free (GF) animals^17^ have aided in unmasking a microbial influence on specific CNS developmental processes. All these models have been invaluable in uncovering an essential role of the gut microbiota in establishment of normal adult stress responsiveness and influencing emotional behaviours such as anxiety-related and normal social functioning.^14, 17, 18, 19, 20, 21, 22^ Manipulation of the timing of colonization in GF animals has further highlighted the important role the gut microbiota has during neurodevelopmental time windows that are critical for establishing normal CNS functions in adulthood.^23, 24^ We thus hypothesized that the absence of microbiota may result in altered transcriptional regulation of the PFC. To this end, we therefore conducted unbiased genome-wide RNA sequencing in the PFC of conventional (CON), GF and GF colonized (exGF) mice to investigate alterations in the PFC transcriptome. Our data uncovered a heretofore-undescribed role for the microbiome in the regulation of myelination.

## Materials and methods

### Animals

GF and CON Swiss Webster breeding pairs were obtained from Taconic (Germantown, NY, USA). GF mice were housed in gnotobiotic isolators under a strict 12-h light/dark cycle in cages of 4–5 animals. On postnatal day 21, just after weaning, exGF mice were taken from isolators and housed next to CON mice. CON and exGF mice were housed in the standard animal unit, which allows for exGF mice to be exposed to environmental microbes resulting in colonization. CON mice are similarly housed under regulated conditions (temperature 20–21 °C, 55–60% humidity), under the same 12-h light/dark cycle and housed 4–5 per cage. Autoclaved, pelleted diets were the same for all animal groups (Special Diet Service, Essex, UK, product code 801010). Animals were culled at week 10 for follow-up experiments. All experiments were approved by the Ethics Committee of University College Cork and the Irish Department of Health authorities.

### Experimental design

F_1_-generation offspring GF, exGF and CON mice were culled in adulthood at 10 weeks. Brains were collected for quantitative real-time PCR (qRT-PCR), for protein analysis and for transmission electron microscopy. For full detailed experimental flow with relevant animal number per experiment, see Supplementary Figure S1.

### Tissue extraction and RNA sequencing

Several key brain regions (amygdala, PFC, striatum, cerebellum, hippocampus and frontal cortex) were rapidly hand-dissected and stored at 4 °C in RNAlater (Sigma-Aldrich, Wicklow, Ireland) for 24 h followed by storage at −80 °C until tissue processing. RNA sequencing was performed as described previously.^25^ For details, see Supplementary Methods.

### Quantitative real-time PCR

RNA was reverse transcribed using high-capacity cDNA reverse transcription kit (Thermo Fisher Scientific, Waltham, MA, USA) in a G-storm thermocycler (G-storm, Surrey, UK). Gene expression was analysed using TaqMan Gene Expression Assays (Supplementary Table S1) on an AB7300 system (Applied Biosystems, Thermo Fisher Scientific). Expression levels were calculated as the average of three replicates for each biological sample from all three groups (*n*=12 per group) relative to β-actin expression. Fold changes were calculated using the ΔΔC_t_ method.^26^ For statistical analysis one-way analysis of variance with Fishers least significant difference *post hoc* test was carried out for each gene. A *P*-value <0.05 was considered statistically significant.

### Protein extraction and western blot

Total protein was extracted using a commercially available mirVana PARIS RNA and Native Protein Purification kit (Thermo Fisher Scientific). Protein levels were detected using appropriate primary antibody dilutions against MOG (1:1000; sourced from Abcam, ab109746) and secondary antibody (Alexa 594-conjugated antibody to rabbit, 1:10 000, Life Technologies, Thermo Fisher Scientific). Primary antibody against alpha tubulin (sourced from Sigma-Aldrich, Cat# T5168) was used to determine equal protein loading. Quantification of protein bands was analysed using Image Studio Lite (LI-COR, Lincoln, NE, USA). Significance was determined by a one-way analysis of variance.

### Transmission electron microscopy

Transcranial perfusion was carried out on six male mice (*n*=3 per group), with 4% paraformaldehyde followed by PFC dissection. Following post-fixation in osmium tetroxide and dehydration in ascending ethanol series followed by propylene oxide, the samples were embedded in Araldite resin (Agar Scientific, Essex, UK). For each specimen, semi-thin (0.5 μm) and thin (70–90 nm) sections were obtained from polymerized blocks using a Reichert-Jung Ultracut E ultramicrotome (Leica-Microsystems, Wetzlar, Germany). Semi-thin sections were stained with toluidine blue and examined using a light microscope. Thin sections from selected areas of the trimmed blocks were made and collected on formvar-coated copper grids (Agar Scientific). Thin sections were double contrasted with 2% uranyl acetate and Reynolds lead citrate stain, and examined using a Jeol 2000FXII transmission electron microscope (JEOL, Peabody, MA, USA), operated at 80 kV. Electron micrographs were obtained of areas of interest with a Megaview-III digital camera and AnalySIS software (EMSIS, Münster, Germany). A minimum of 50 myelinated axons were measured per animal.

### Statistics

All statistics used for analysis of differential gene expression were according to the description of R package *DESeq2* with default parameters. Multiple-testing corrections were performed for all analyses, the adjusted *P*-value (*P*_adj_) was calculated using the Benjamini–Hochberg procedure (also known as false discovery rate), also *P*-values reported for functional annotation and enrichment analysis tools were multiple-testing-adjusted *P*-values (FRD). A *P*_adj_<0.1 was considered significant for these analyses. A detailed description of statistics used for RNA-seq data analysis can be found in Supplementary Methods. Graphpad Prism (v5) (GraphPad Software, La Jolla, CA, USA) was used for plotting, Grubbs method^27^ was used to test for outliers and statistical testing for qRT-PCR was carried out on SPSS v21. Statistical significance of overlaps between gene lists was computed using Fischer's exact test (http://nemates.org/MA/progs/overlap_stats.html).

## Results

### Changes in PFC transcriptome and marked upregulation of genes related to myelination in GF animals

**Figure 1:**
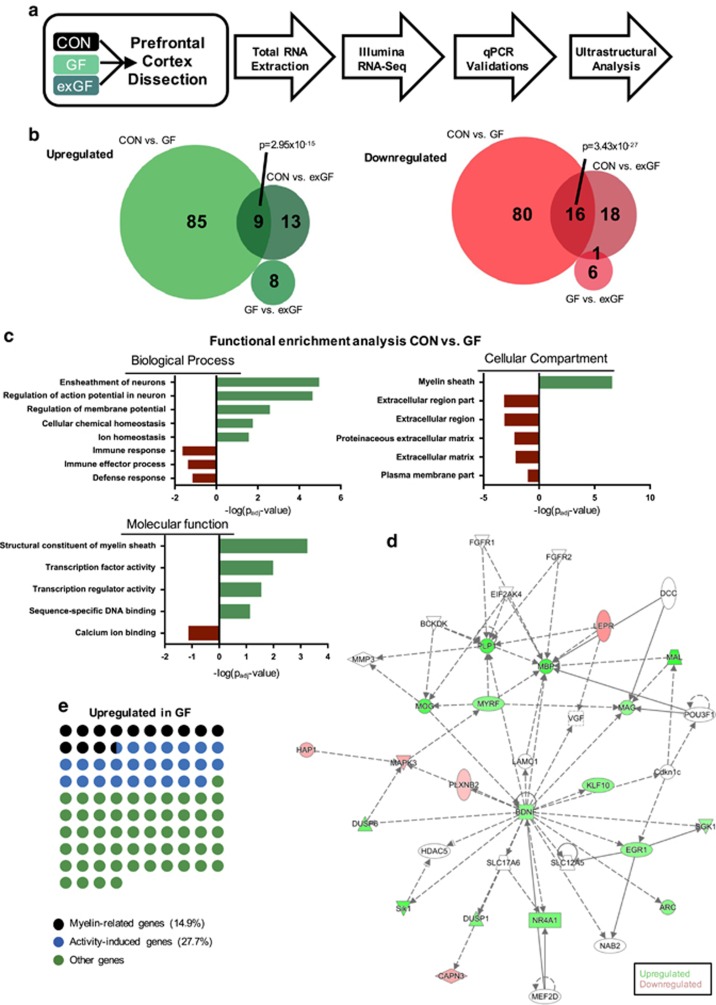
Differential gene expression in the prefrontal cortex of conventional (CON) and germ-free (GF) raised mice highlights myelin pathways to be significantly different. (**a**) Schematic of experimental design. (**b**) Venn diagrams representing overlaps of differentially expressed genes between experimental groups. A total of 236 genes were found to be different between groups. *P*-values indicate significant overlaps as determined by Fisher's exact test. (**c**) Functional enrichment analysis revealed significantly enriched Gene Ontology terms in GF condition associated with myelin sheath formation and regulation of action potentials amongst upregulated genes. Enrichment of downregulated genes highlights immune and defence mechanisms. (**d**) Gene network representing interactions and upstream regulators of genes affected in GF mice compared with CON mice. Network highlights interactions of upstream transcription factors regulating myelin component genes. (**e**) Fractions of upregulated genes in GF that are known to regulate myelination or are myelin component genes. Each dot represents one of 94 genes upregulated in GF mice.

We performed unbiased deep sequencing of polyA-enriched RNA extracted from the PFC of male conventionally raised (CON) mice, GF mice and an additional group of GF mice that were colonized post weaning (postnatal day 21; exGF; Figure 1a). Analysing gene expression amongst all three groups by pairwise comparisons, we found 221 genes to be significantly differentially expressed in GF, or exGF animals relative to CON controls (Figure 1b and Supplementary Table S2). Of these, a total of 190 genes were differentially expressed between CON and GF samples, and a smaller proportion of genes^28^ showed differential expression in exGF mice (Supplementary Table S2). Comparing GF and exGF directly, there were only 15 genes differentially expressed (Figure 1b). A significant overlap of genes changed in both, GF and exGF conditions was noted, (Figure 1b), suggesting that certain cellular regulatory functions were irreversibly affected by the absence of a microbiota during early life.

To further investigate cellular functions linked to differentially expressed genes, we performed gene ontology (GO) enrichment analysis. We found a strong overrepresentation of genes involved in myelination among genes upregulated in GF mice, reflected by several enriched related GO terms in all three GO categories (Figure 1c and Supplementary Table S3). In addition, we found significant enrichment of genes implicated in transcriptional regulation (Figure 1c and Supplementary Table S3).

Next, we examined whether differentially regulated genes are functionally connected using the Ingenuity Pathway Analysis tool (IPA, QIAGEN, Redwood City, CA, USA, www.qiagen.com/ingenuity). The resulting network was centred around brain-derived neurotrophic factor, a well-known regulator of neuronal plasticity, but also the myelin regulatory factor, predicted to have a central part in driving expression of several myelin component genes (Figure 1d). Moreover, network analysis revealed interaction of several activity-induced immediate early genes as well as components of the MAP-kinase pathway, which has been shown to interact with myelin regulation in adulthood through myelin regulatory factor^29^ (Figure 1d).

**Figure 2:**
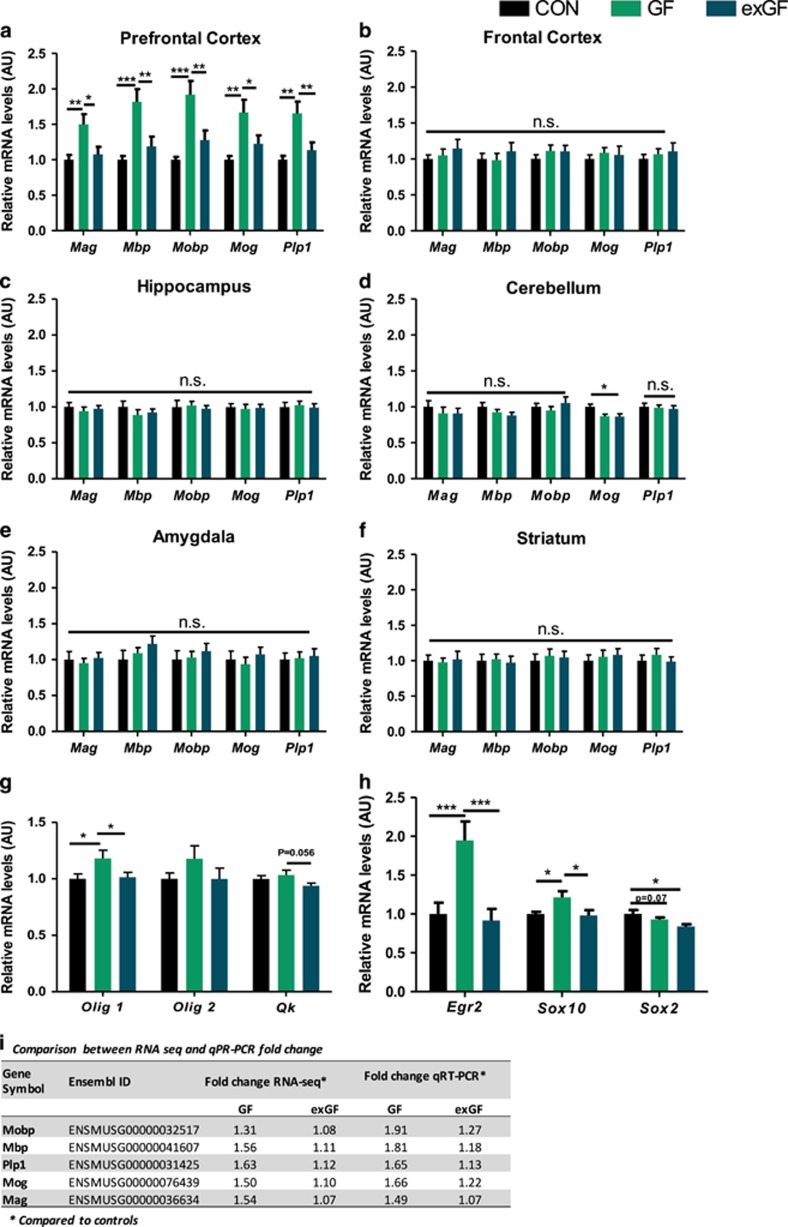
Quantitative real-time PCR (qRT-PCR) validations of RNA-seq data within various brain regions of selected myelin component genes were found to be brain region specific. Germ-free status in mice results in increased myelin gene expression only in the PFC, which was normalized in exGF mice. (**a**–**f**) qRT-PCR of myelin gene transcripts and regulatory factors in the prefrontal cortex, frontal cortex, hippocampus, cerebellum, amygdala and striatum. Bar graphs indicate average values in 12 mice per group after β-actin normalization relative to average control levels. (**g**) Significant changes in oligodendrocyte-specific genes. (**h**) Changes in known genes involved in regulation of myelination. (**i**) Table representing RNA-seq and qRT-PCR fold change for individual myelin component genes used for RNA-seq validation. Fold change is in comparison with the control group. (**a**, **g**, **h**) Prefrontal cortex, (**b**) frontal cortex, (**c**) hippocampus, (**d**) cerebellum, (**e**) amygdala and (**f**) striatum. Data graphed as ±s.e.m. *P<0.05; **P<0.01; ***P<0.001.

Therefore, and as our enrichment analysis suggested both increased expression of myelin components, as well as activation of pathways induced by neuronal activity, we then further manually scrutinized the list of upregulated genes for involvement in myelin-related processes. We found a total of 14 out of 94 genes upregulated (14.9%) in the PFC of GF mice were directly linked to myelination. A total of 26 genes (27.7%) were associated with neuronal activity as determined by cross-referencing with gene expression data from the literature.^30, 31^ Using a more stringent *P*-value cutoff (*P*_adj_⩽0.05), 12 (19%) and 17 (27%) out of 63 upregulated genes were myelin-associated or associated with neuronal activity, respectively (Supplementary Table S2). The oligodendrocyte myelin glycoprotein (*Omg*) gene was found to be involved in both processes. Together these two processes explained more than 40% (46% for *P*_adj_⩽0.05) of the upregulated genes (Figure 1e), indicating a strong, orchestrated transcriptional regulation pattern. Notably, we did not find any of these genes to be differentially regulated in exGF mice, suggesting that myelin-related and activity-induced gene changes are dynamically influenced by the presence of host-associated microbes. Using highly sensitive, specific qRT-PCR assays for five myelin component genes, which all encode for key structural proteins that contribute to the integrity and function of the myelin sheath, we confirmed overexpression in the PFC of GF animals. qRT-PCR values closely mirrored RNA sequencing thereby validating genome-wide results (Figure 2i). Interestingly, colonization post weaning normalized transcript abundance to CON levels in exGF mice, indicating that targeting the microbiota during critical periods of synaptic reconstruction (early adolescence post weaning) had significant effects on myelination at the transcriptional level.

### Overexpression of myelin component genes is region- and sex-specific

Next we tested whether the upregulation of myelin genes could also be detected in other brain areas. Importantly, elevated transcription of myelin component genes in GF mice was confined to the PFC as no changes in mRNA levels were found for the cerebellum, amygdala, hippocampus, striatum or frontal cortex (Figures 2b–f), demonstrating a brain region-specific effect of colonization status. Following from this, we wanted to appraise whether changes in oligodendrocyte activity could underpin changes in myelin component gene transcripts by investigating changes in oligodendrocyte-specific genes. We observed a slight increase in *Sox10* and *Olig1* expression, transcription factors and upstream activators of increased myelin gene transcription involved in promoting terminal differentiation of oligodendrocyte progenitor cells to mature oligodendrocytes. (Figures 2g and h). Coinciding with increased *Sox10*, a twofold increase in *Egr2* expression, an immediate early response gene and positive regulator of myelination interacting with *Sox10*, was found in GF mice and subsequently normalized to control levels in exGF mice (Figure 2h). We also assessed myelin gene expression levels in female GF mice and found no increase in myelin gene expression (Supplementary Figures S2a–i).

### Absence of microbiota results in hypermyelinated axons in the PFC

**Figure 3:**
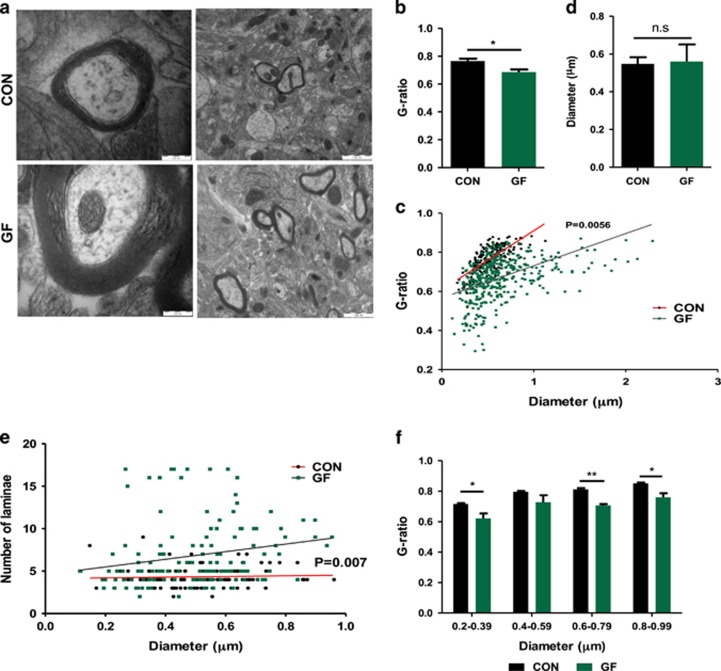
Increased myelin sheath thickness in the prefrontal cortex (PFC) of male germ-free (GF) mice. (**a**) Electron micrographs of axons in the PFC of conventionally raised (CON) and GF mice. Scale bars=200 and 1000 nm. (**b**) Average *g*-ratio per animal in the PFC. (**c**) Scatter plot of *g*-ratio values in the PFC in CON (*n*=187 axons) and GF (*n*=390 axons) against axon diameter. (**d**) Average axonal diameter per animal. (**e**) Scatter plot of axon diameter against the number of lamina counted for that individual axon. (**f**) *g*-ratio for individual axonal population based on diameter range. Bar graphs shown as mean±s.e.m. NS (not significant) *P*>0.05; *P<0.05; **P<0.01.

**Figure 4:**
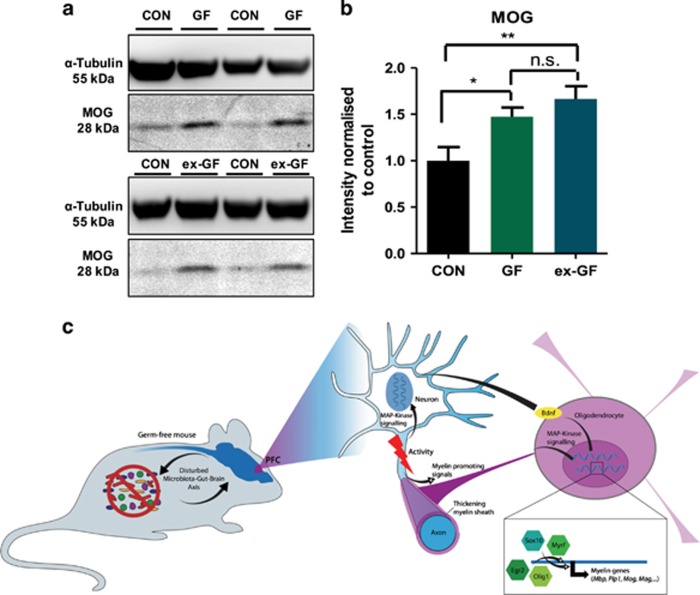
Increased myelin protein in male germ-free (GF) and exGF mice. (**a**) Western blot analysis for MOG in the prefrontal cortex (PFC) of conventional (CON), GF and exGF. (**b**) Quantification of protein concentration was normalized to β-III-tubulin and expressed relative to control levels. (**c**) Schematic representation of findings highlighting the transcriptional network driving increased myelination. Bar graph data is shown as mean±s.e.m. *P<0.05; **P<0.01.

As upregulation of a large number of myelin regulatory and myelin component genes suggested increased formation of myelin in the PFC of GF animals, we next tested whether this gene regulatory effect would correlate with altered oligodendrocyte function, that is, axonal myelination in the PFC. Using transmission electron microscopy, we analysed myelinated axons and found that male GF mice displayed thicker myelin sheaths in the PFC as indicated by a significantly lower *g*-ratio (quantification of myelin thickness relative to axonal diameter, mean 0.76±0.016 and 0.68±0.017; *P*<0.05; Figures 3a–c). We compared the calibre of axons and found no size differences between CON and GF mice, indicating that the decreased *g*-ratio was not due to pathological shrinkage of axon calibre (Figure 3d). Furthermore, increased myelin sheath thickness coincided with a strong trend towards higher lamina number and thicker myelin diameter in GF mice (Supplementary Figures S3a and b). Longitudinally sliced axons were excluded from *g*-ratio analysis, but random sampling analysis of longitudinally cut axons revealed that hypermyelination was uniform along the internodal region (Supplementary Figure S3c). When representing *g*-ratio as diameter against lamina number, CON animals showed consistent lamina number (mean 4.4) independent of axonal diameter whereas, GF mice show greater variability in the number of lamina per axon, indicating that increased thickness could be a result of increased number of wrappings (Figure 3e). As selection of the axons to myelinate can be based on axonal calibre we compared *g*-ratios of myelinated axons in GF and CON mice, plotted as a function of their respective axonal diameters, which revealed that hypermyelination was not more pronounced in smaller axons than in larger axons, again indicating the observed hypermyelination is independent of axonal calibre (Figure 3f). In order to evaluate whether transcriptional levels of myelin component genes mimicked protein levels, we conducted western blot analysis for the MOG protein. Importantly, increased mRNA levels were paralleled by increased protein in the PFC of GF mice (Figures 4a and b). However, normalization of transcript levels for this gene following colonization in exGF mice did not translate to a reduction in protein levels, suggesting that colonization post weaning could not reverse increased myelin protein abundance, likely accumulating before weaning.

## Discussion

There is a growing appreciation of the ability of host–microbe interactions to modulate brain networks related to psychiatric disorders. The most consistent finding related to the abnormal patterns noted in GF animals linked to anxiety and sociability behaviours.^17, 19, 20, 21, 22^ Our results reveal highly coordinated differential programme of gene expression in the PFC in these animals, a key brain region in anxiety and social behaviour, which may contribute to the GF behavioural phenotype. Surprisingly, a large proportion of significantly upregulated genes (~15%) were found to be related to myelination. These included well-known genes coding for structural components of the myelin sheath but also the major myelin regulatory factor *Myrf* and other oligodendrocyte lineage-specific genes, suggesting an orchestrated upregulation of myelin formation. Importantly, the observed alterations showed an exquisite regional specificity as we did not detect the upregulation of myelin-related genes in any of the other investigated brain regions. Although regional specificity in myelin regulation remains a unexpected finding, regional effects of transcriptional regulation by the microbiome is in agreement with previous findings in the literature.^17, 25^

Analysis of the PFC ultrastructure revealed that the marked upregulation of myelin-related gene expression resulted in increased myelin sheath thickness as indicated by an overall decrease in *g*-ratio. It has been shown that myelination of the various cortical areas follows a certain order, where primary sensory and motor areas myelinate before the association areas, including the PFC. Moreover, neuroimaging studies have indicated that there is continual myelination in the PFC until the third decade of life.^32^ In addition, preclinical studies have shown that myelination in the PFC undergoes plastic changes, influenced by environmental stimuli mediated by increased electrical activity in axons.^33^ As such, social deprivation in neonatal and juvenile mice results in defective development of myelin^34, 35^ indicating that myelination in the PFC is extremely plastic during critical neurodevelopmental phases.

In our study we also see evidence of myelin-related plasticity, as colonizing GF mice (exGF) counteracts the altered increase in myelin-related transcripts. The plastic nature of PFC myelination has also been observed in adult mice following prolonged periods of social isolation. This protocol resulted in the development of a phenotype marked by social withdrawal, which coincided with changes in myelination only in the PFC.^36^ Subsequent group-housing reverted social deficits and reinstated normal levels of myelin.^36^ This earlier study indicates that myelination contributes to functional adult brain plasticity and that PFC myelination can be dependent on appropriate social experience in mice. Taken together with our results, a complex picture emerges of a malleable interaction between the gut microbiome, social behaviour and PFC myelination across the lifespan.

Our transcriptome and qRT-PCR data point to a normalizing effect of introducing a microbiota post weaning. The sustained increase in MOG protein levels in exGF mice (Figures 4a and b) could be explained by the timing of colonization post weaning. As such, previous studies have highlighted that colonizing GF mice with either a CON microbiome or by mono-association between weeks 3 and 6 can partially revert the altered hypothalamic–pituitary–adrenal- and anxiety-related phenotype associated with these animals.^18, 20^ However, in mice formation of myelin occurs earlier, around postnatal day 10 (ref. 37), with a capacity to continue into adulthood in a brain region-specific manner. As our exGF mice were still sterile during this important time window, colonization commencing at P21 may not be sufficient to normalize this heightened myelin protein accumulation. Indeed, the concept of critical overlapping neurodevelopmental and early-life microbial colonization windows is consistent with the literature in this area.^24^ The dynamic nature and continued ability of the PFC to undergo marked changes in myelination even in adulthood may, at least in part, explain why the PFC is more susceptible to a microbial influence on myelination than the other brain regions tested. Further studies manipulating the timing of colonization (colonization before weaning) will be required to investigate the potential dynamic nature of microbes influencing myelination and to whether the observed increase in protein in our exGF can be normalized under such conditions.

Dynamic regulation of myelin gene expression is poorly understood. However, a growing number of studies show that myelination is dependent on neuronal activity.^33, 38, 39, 40^ This strongly agrees with our data and indicates that neuronal activity is indeed increased in the PFC of GF mice, as suggested by a marked upregulation of a large number of genes known to be expressed in response to raised neuronal activity. Our present study suggests that increased myelin gene expression may be a result of heightened baseline activity in the PFC. As the PFC receives glutamatergic input from the amygdala, this is in line with our previous finding that the amygdala also appears to be hyperactive in GF mice.^25^ Our interaction network analysis is consistent with the view that myelination is regulated by plasticity-promoting extracellular signals and MAP-kinase signalling via the key transcription factor myelin regulatory factor.^29, 41^ Further support for a plasticity-dependent mechanism of hypermyelination in the PFC under GF conditions comes from the fact that neurotrophic signalling by brain derived neurotrophic factor, which we also found to be upregulated in GF mice in this data set, can promote myelination.^42, 43^

Our data also indicate that *Myrf* is a major driver of the observed myelin gene increase. *Myrf*, in comparison with other myelin-regulating transcription factors, is insufficiently described as it has only recently been discovered as the major player in the establishment of appropriate functional myelination. It is critical in the generation of CNS myelination during development and for myelin maintenance in adulthood as ablation of *Myrf* in mature oligodendrocytes in the adult CNS results in severe demyelination and rapid downregulation of myelin-related genes such as *Plp* and *Mbp*.^44^ A recent study further investigated its role in myelination and found that *Myrf* physically interacts with *Sox10* (ref. 45), a known transcription factor that regulates, along with *Egr2*, peripheral nervous system myelination. *Sox10* itself, via interaction with *Myrf*, is necessary for terminal differentiation of oligodendrocytes in the CNS. Though neither *Sox10* nor *Egr2* did appear in our list of differentially regulated genes (Supplementary Table S4), we found that there was a modest increase in the expression of *Sox10* (~1.2-fold) and *Egr2* (~2-fold) in GF mice, which was not evident when expression levels were compared between CON and exGF mice.

*Sox10* induction cannot be triggered alone, and the addition of intrinsic and extrinsic factors is required to drive oligodendrocyte maturation.^46, 47^ Previous studies indicate that chromatin remodelling by *Olig2*, an oligodendrocyte-specific transcription factor, has the capacity to alter accessibility of key regulating gene such as *Mryf* at the onset of terminal differentiation.^48^ We observed a significant increase in *Olig1* but not in *Olig2*. *Olig1*, similar to *Olig2*, has also been reported to interact with *Sox10* and drive the expression of key myelin genes such as *Mbp*.^49, 50^ As *Sox10* activity is present at all stages of oligodendrocyte development and *Myrf* appears to be essential in the final steps of oligodendrocyte development, it may be at this point of terminal differentiation that GF mice have altered oligodendrocyte activity, which is further supported by the presence of excessive laminae in our study (Figure 4c).

It has recently been shown that the microbiota contributes to microglia homeostasis, with GF mice showing marked global deficits in microglia.^51, 52^ Interestingly, this study did not find altered numbers of oligodendrocytes in GF mice. Although the authors did not specifically look at the PFC nor was myelination investigated, together these data render it unlikely that the altered oligodendrocyte-specific transcriptional profile observed here results from an increased number of oligodendrocyte cells. In contrast, higher lamina counts and increased myelin thickness together with orchestrated upregulation of myelin gene regulators rather reflect altered activity and functionality of oligodendrocytes in GF mice.

Our results were unexpected as, in stark contrast to a large body of literature on demyelination, studies on the consequences of hypermyelination on brain function in health and disease are essentially non-existent. Changes in myelin abundance have been reported in many neuropsychiatric disorders, and links to behavioural outcomes of these disorders have been established in animal models.^33^ For example, large genome-wide analyses of the PFC of schizophrenic patients have remarkably highlighted abnormal regulation of genes related to myelination.^48, 53^ Most of these studies report reduced myelin gene expression and white matter volume, with neuroregulin 1 (*NRG1*), a schizophrenia susceptibility gene,^54, 55^ expression increased. Transgenic mice overexpressing *NRG1*, previously linked to hypermyelination in these mice, displayed tremor, impaired performance on rotarod and decreased prepulse inhibition performance.^56, 57^

Although our study further contributes to the mechanistic understanding of microbial effects on the largely descriptive literature on behavioural alterations in GF animals, the exact mechanisms underpinning the control exerted by the microbiota over myelination in the CNS remain speculative. Known routes of gut–brain communications include neuroactive microbial metabolites,^28^ as well as signalling through the vagus nerve.^6, 55^ Importantly, as the main central relay for incoming information from the vagus nerve, the nucleus tractus solitarius has an extensive network of projections, including the parabrachial nucleus, which further projects to the PFC and also the amygdala, a region we have previously shown to be susceptible to microbial transcriptional regulation.^1, 25^ In addition, microbes directly affect the immune system resulting in alterations in the circulating levels of pro-inflammatory and anti-inflammatory cytokines that can affect oligodendrocyte development.^58, 59^

Although there have been some reports of a functional role of the intestinal microbiota in development of experimental autoimmune encephalomyelitis^12^ demonstrating that GF animals have a higher resistance to experimental autoimmune encephalomyelitis development^60^ and the potential for the gut microbiota as a risk factor for inflammation-mediated CNS demyelination,^11^ this report, to our knowledge, represents the first example of direct myelin regulation in the CNS by the microbiome. This is evident at both the transcriptional and ultrastructural levels in adulthood. Furthermore, the gene expression changes were brain region- and sex-specific, and amenable to plastic modulation by introduction of a microbiota post weaning. Several studies in GF mice have demonstrated predominantly sex-specific brain effects, with males showing more robust alterations at the level of the CNS.^20^ Further studies are needed to ascertain why GF females do not display many of the altered phenotypes of their male counterparts, and research exploiting the GF model should take this into account.

In conclusion, the present study indicates that appropriate cortical myelination relies on the presence of a functional microbiota during critical windows of neurodevelopment. In-depth analysis of transcriptome data further suggested that hypertrophic neuronal signalling in the PFC of GF mice might be underlying the observed increase in myelination. Our data indicate that the microbiome is critical for appropriate cortical myelination. Our results further highlight the microbiota as a viable therapeutic target in psychiatric disorders and may allow to develop strategies to promote remyelination in myelination diseases. Owing to what we have demonstrated with GF animals, future studies utilizing alternate models of microbiota depletion such as chronic antibiotic treatment may further add to our understanding of the influential relationship of the microbiota on cortical myelination. Studies utilizing approaches such as monocolonization in either GF or microbiota-depleted animals using antibiotics would allow deciphering whether specific bacterial strains have the capacity to normalize the observed altered myelination patterns in these animals. In addition, subsequent studies aiming to mechanistically understand the influential role of the gut microbiota on myelination should examine whether certain bacterial metabolites could be underlying myelin changes or what role the vague nerve has in the communication between the gut bacteria and CNS myelination (that is, whether signalling along the vagus nerve is required).
