# Supplementary material for: Regulation of prefrontal cortex myelination by the microbiota
Source: Transl Psychiatry. 2016 Apr 5;6(4):e774–. doi: 10.1038/tp.2016.42 (PMC4872400; doi:10.1038/tp.2016.42)
Supplement: Supplementary Figure 1 [file tp201642x1.pdf]

Supplementary figure 1: Experimental flow chart

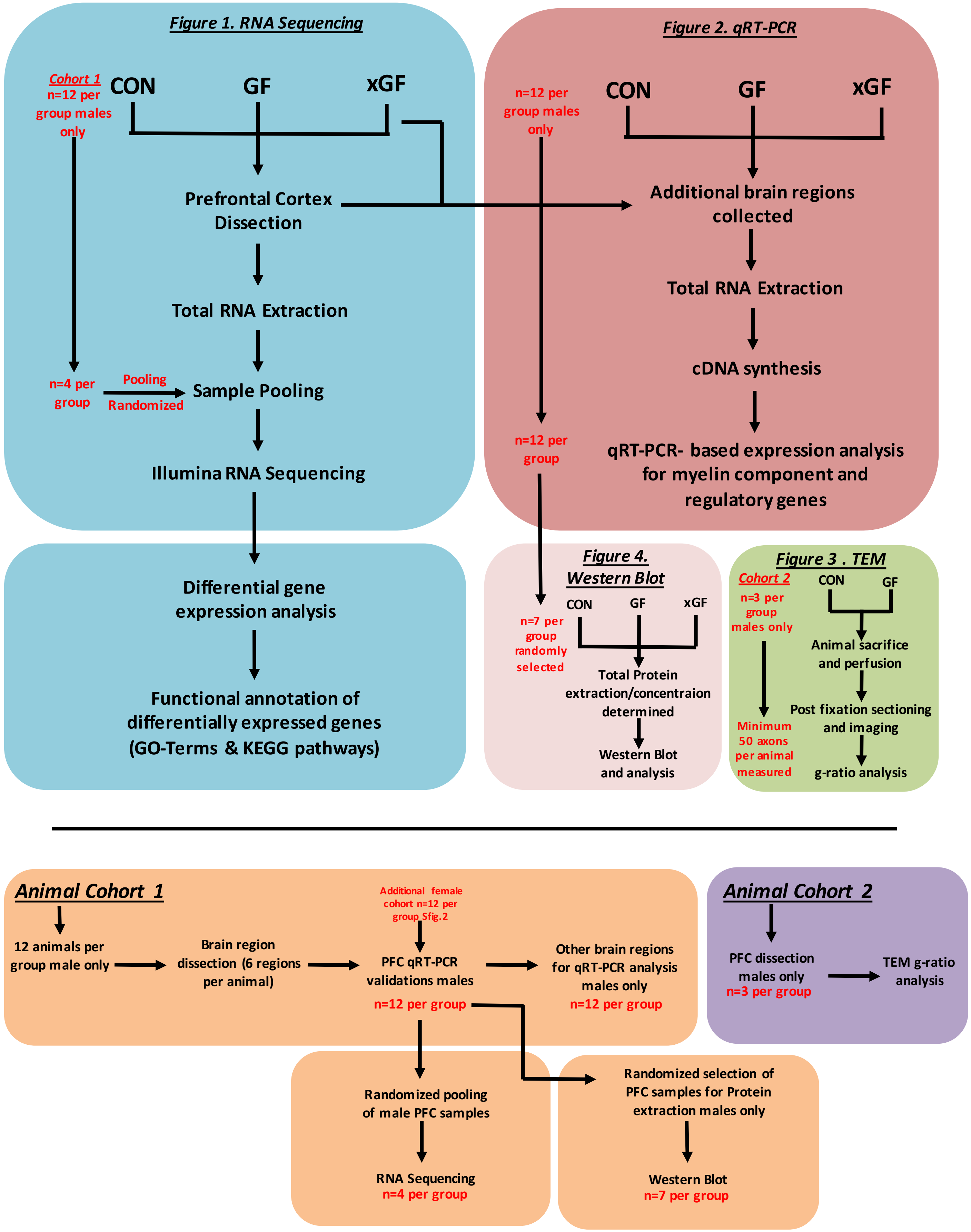

Figure S1: Experimental flowchart. Graphical depiction of the animal number and usage for each individual experiment. Each Panel represents the outcome of tissue used for each of the individual main figure.

Supplementary figure 2: Female mice

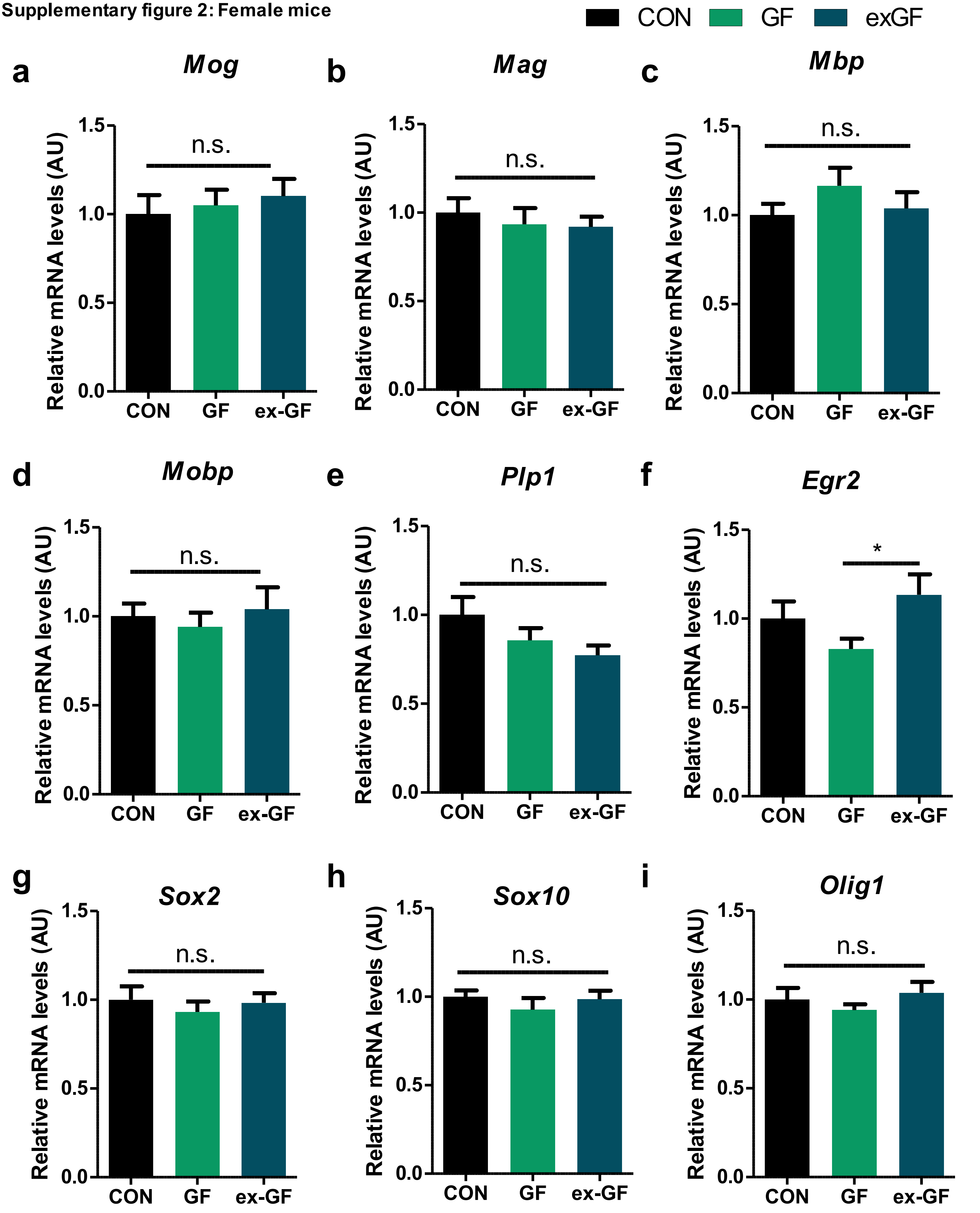

Figure S2: mRNA expression levels of myelin component and upstream myelin regulatory genes in the PFC of <u>female</u> CON, GF and exGF mice. No male-equivalent changes in myelin component mRNA levels were found in the PFC of female GF and exGF mice. **(a-i)** qRT-PCR of myelin component gene transcript and myelin regulating transcription factors in the PFC. Bar graphs indicate average values of 12 animals after *β-actin* normalization relative to average control levels ($p<0.05$ *). Data graphed as +/- SEM.

Supplementary figure 3: Laminae and logitudinally cut axons

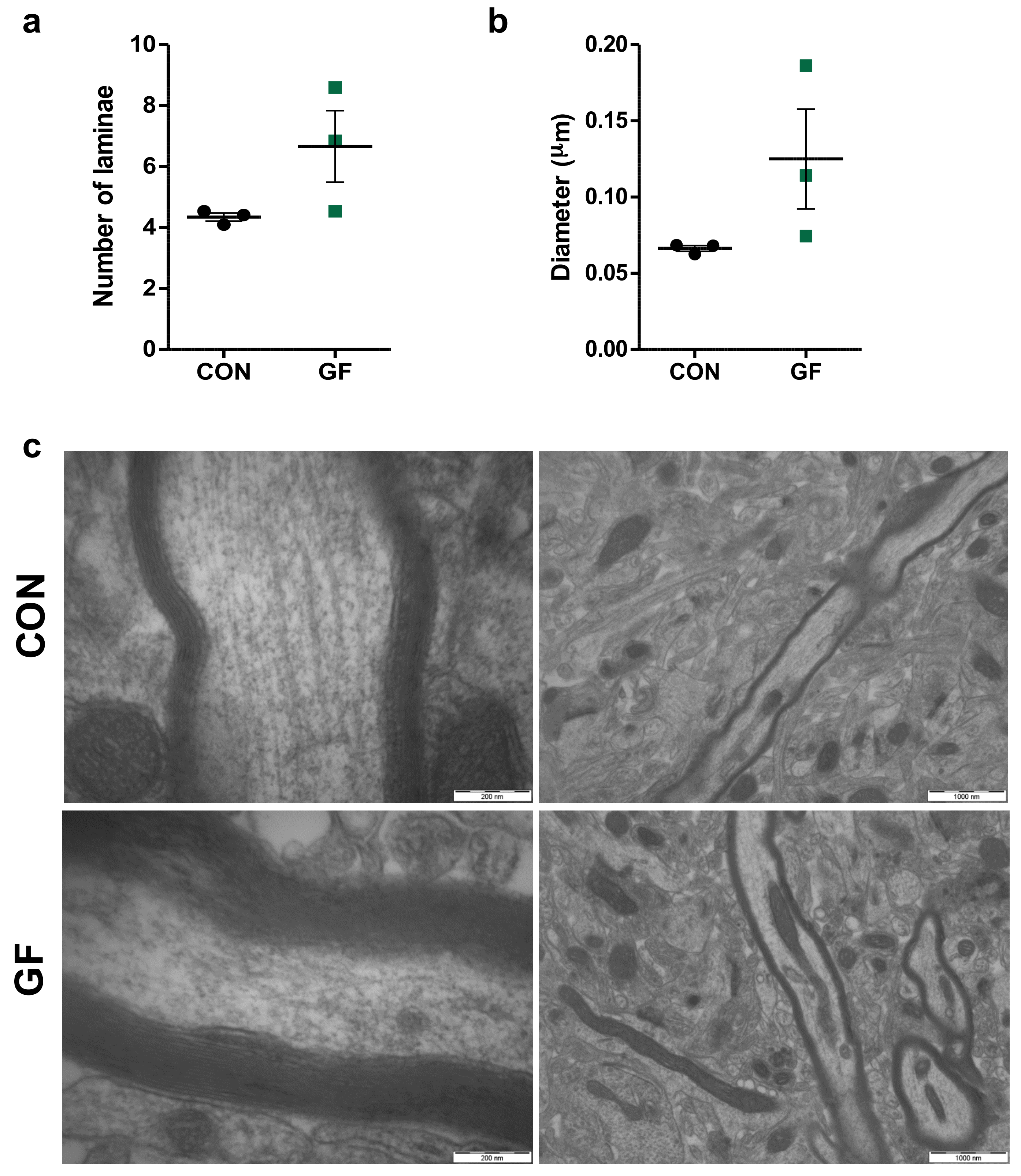

Figure S3: Increased g-ratio coincides with stronger trend to higher lamina number and myelin diameter. (a) Average lamina number per animal in the PFC of GF and CON mice. **(b)** Average myelinated diameter per animal **(c)** Electron micrograph of longitudinally sliced axons in the PFC of CON and GF mice. Scale bar 200nm and 1μm. Bar graph data is shown as mean +/- SEM. **n.s.** indicated p>0.05; n=3 animals per group; n=>50 axons per animal (CON n=187 axons; GF n=390 axons).
