# Supplementary material for: Regulation of prefrontal cortex myelination by the microbiota
Source: Transl Psychiatry. 2016 Apr 5;6(4):e774–. doi: 10.1038/tp.2016.42 (PMC4872400; doi:10.1038/tp.2016.42)
Supplement: Supplementary Information [file tp201642x2.doc]

**RNA sequencing**

RNA was extracted using commercially available mirVana^TM^ total RNA extraction kit (Ambion/life technologies) and DNase treated (Turbo DNA-free, Ambion/life technologies) according to manufacturer’s protocol. RNA was high quality with an RNA integrity number (RIN) value for all samples above 8 (Bioanalyzer, Agilent). Prefrontal cortex samples were then randomly pooled within each group by combining equal amounts of RNA from two to three animals resulting in a final four samples per group sent for RNA sequencing at Exiqon (Vedbaek, Denmark), where library preparation, paired-end sequencing (2x100bp) and Fastq-file generation was conducted on an Illumina NextSeq500 sequencer. The data discussed in this publication have been deposited in NCBI's Gene Expression Omnibus (Edgar et al., 2002) and are accessible through GEO Series accession number GSE66828 (http://www.ncbi.nlm.nih.gov/geo/query/acc.cgi?acc=GSE66828).

***Quality control and mapping to reference genome***

Reads were filtered for quality and trimmed using Trimmomatic (v0.32) (1) using the following non-default parameters: *AVGQUAL*: 20; *SLIDINGWINDOW*: 4:20; *LEADING*: 10; *TRAILING*: 10; *MINLEN*: 60. Alignment to the mouse reference genome (GRCm38.p3) was performed with STAR aligner (v2.4.0f1) (2). Ensembl database release 78 served as input for gene models.

***Differential gene expression and GO-Term enrichment analyses***

Ensembl release 78 was used for read counting for each gene using HTSeq-Count (v0.6.0) (3) with the following non-default parameters: -s: no; -r: pos; -q –f bam –m intersection-nonempty. Differential gene expression was determined using the DESeq2 R-package (v1.6.2) (4,5) with default parameters on pairwise comparisons of all possible group combinations. An adjusted p-value ≤ 0.1 (Benjamini-Hochberg method) was considered significantly differentially regulated. Raw and processed original data have been deposited in NCBI's Gene Expression Omnibus (6) and and are accessible through GEO Series accession number GSE75816 (http://www.ncbi.nlm.nih.gov/geo/query/acc.cgi?acc=GSE75816). Differentially expressed genes were then analysed for enrichment of GO-Terms using the DAVID Bioinformatic Resources (v6.7) (7). A network of molecular interactions was generated with QIAGEN’s Ingenuity® Pathway Analysis (IPA®, QIAGEN Redwood City, [www.qiagen.com/ingenuity](http://www.qiagen.com/ingenuity)).

**References**

1. Bolger AM, Lohse M, Usadel B. Trimmomatic: a flexible trimmer for Illumina sequence data. Bioinformatics. 2014 Aug;30(15):2114–20.

2. Dobin A, Davis CA, Schlesinger F, Drenkow J, Zaleski C, Jha S, et al. STAR: ultrafast universal RNA-seq aligner. Bioinformatics. 2013 Jan;29(1):15–21.

3. Anders S, Pyl PT, Huber W. HTSeq--a Python framework to work with high-throughput sequencing data. Bioinformatics. 2015 Jan;31(2):166–9.

4. Love MI, Huber W, Anders S. Moderated estimation of fold change and dispersion for RNA-seq data with DESeq2. Genome Biol. 2014;15(12):550.

5. Anders S, Huber W. Differential expression analysis for sequence count data. Genome Biol. 2010;11(10):R106.

6. Edgar R, Domrachev M, Lash AE. Gene Expression Omnibus: NCBI gene expression and hybridization array data repository. Nucleic Acids Res. 2002 Jan;30(1):207–10.

7. Huang DW, Sherman BT, Lempicki RA. Systematic and integrative analysis of large gene lists using DAVID bioinformatics resources. Nat Protoc. 2009 Jan;4(1):44–57.
